# Induction chemotherapy‐based organ‐preservation protocol improve the function preservation compared with immediate total laryngectomy for locally advanced hypopharyngeal cancer—Results of a matched‐pair analysis

**DOI:** 10.1002/cam4.6354

**Published:** 2023-07-19

**Authors:** Yifan Yang, Ling Feng, Qi Zhong, Yang Zhang, Zhigang Huang, Shurong Zhang, Shuling Li, Junmao Gao, Lizhen Hou, Hongzhi Ma, Shizhi He, Qian Shi, Meng Lian, Yanming Zhao, Xixi Shen, Jiaming Chen, Lingwa Wang, Haiyang Li, Shaoshi Chen, Jiaqi Xu, Ru Wang, Jugao Fang

**Affiliations:** ^1^ Department of Otorhinolaryngology, Head and Neck Surgery, Beijing Tong Ren Hospital Capital Medical University Beijing China; ^2^ Key Laboratory of Otorhinolaryngology, Head and Neck Surgery Beijing Institute of Otorhinolaryngology Beijing China; ^3^ Department of Oncology, Beijing Tong Ren Hospital Capital Medical University Beijing China; ^4^ Department of Radiology, Beijing Tong Ren Hospital Capital Medical University Beijing China; ^5^ Department of Radiotherapy, Seventh Medical Center General Hospital of the Chinese People's Liberation Army Beijing China

**Keywords:** chemotherapy, hypopharyngeal cancer, laryngectomy, organ preservation, surgery

## Abstract

**Background:**

We performed a paired analysis to compare the therapeutic effect between the induction chemotherapy‐based organ‐preservation approach and immediate total laryngectomy in hypopharyngeal squamous cell carcinoma patients requiring total laryngectomy.

**Methods:**

351 patients who were treated with organ‐preservation approach were compared with 110 patients who were treated with total laryngectomy. The main measures and outcomes were progression‐free survival (PFS), overall survival (OS), and larynx function preservation survival (LFPS).

**Results:**

No statistical difference was observed for 3‐, 5‐, and 10‐year PFS and OS in two groups. In the organ‐preservation group, the 3‐, 5‐, and 10‐year LFPS was 30.7%, 23.3%, and 16.6%, respectively. The LFPS of Stage III > Stage IV, N0 > N1 > N2 > N3, T2 > T3 > T4, CR > PR > SD > PD patients (all *p* values <0.05).

**Conclusions:**

Survival outcomes did not significantly differ between the two groups. The organ‐preservation approach allowed more than 70% of the survivors to retain their larynx function.

## 1 INTRODUCTION

Hypopharyngeal cancer (HPC) is a heterogeneous disease that can involve multiple sites including the pyriform sinus, retropharyngeal wall, and postcricoid region. It accounts for 0.4% of the malignant tumors of the whole body, comprising about 4% of all head and neck cancer cases.^1^ Although HPC is rare, a high incidence of submucosal infiltration, distant metastasis and lymph node metastasis is observed among HPC patients. Due to the complicated anatomical structure of the hypopharynx and the inconspicuous symptom of the early stage, about 70% of patients with HPC present with Stage III or IV disease.^1^, ^2^ In 2020, the number of new cases of HPC was 84,254, and the number of new deaths was 38,599 worldwide in 185 countries, bringing a heavy burden to society.^1^ The traditional treatment strategy for advanced HPC was total laryngectomy. The essential functions of the larynx including breathing, swallowing, and speaking were inevitably destroyed, thus further deteriorating the economic and mental burden of the patient. Recent understanding of the personalized larynx‐preservation strategy has offered new therapeutic possibilities to advanced HPC patients.

Induction chemotherapy (ICT) is an anticancer drug strategy in the initial treatment of cancer patients. Since the 1990s, many landmark studies^3^, ^4^, ^5^ have attempted to investigate the role of ICT in larynx‐preservation treatment. The results of these studies showed that ICT was a larynx‐preservation strategy, which did not negatively affect the survival rate and definitive treatment. ICT was considered as a well‐tolerated mean of selecting HPC patients for an organ‐preservation approach or otherwise have undergone total laryngectomy according to the response. In 2009, two clinical trials^6^, ^7^ supported the superiority of TPF regime. TPF based ICT with was considered a good method to larynx‐preservation with adequate prophylaxis.

However, few studies are comparing classical total laryngectomy to the ICT‐based (TPF regimen) organ‐preservation protocol of HPC patients. Some teams initiated their research only for laryngeal cancer while others considered both larynx and hypopharynx, they would get better performance status because salvage surgery was reputed less morbid for the primary site. Therefore, we conducted a matched‐pair analysis and compared the organ‐preservation and initial surgery treatments concerning overall survival (OS), progression‐free survival (PFS), locoregional control, and larynx function preservation survival (LFPS) in patients with locally advanced HPC.

## 2 MATERIALS AND METHODS

### 2.1 Patients

A total of 461 patients with stage III/IV hypopharyngeal squamous cell carcinoma (HPSCC) treated at Affiliated Beijing Tongren Hospital of Capital Medical University between 2002 and 2022 were reviewed for this retrospective study. The study was approved by the Ethics Committee of Beijing Tongren Hospital, Capital Medical University.

The inclusion criteria include: (1) age range from 18 to 80 years; (2) pathologically proven SCC of the hypopharyngeal, hypopharynx tumors were operable at the first attempt; (3) Stage III/IV resectable HPSCC, treated suitable for only total laryngectomy with partial pharyngectomy; (4) follow‐up was available for at least 6 months or until death. The exclusion criteria include: (1) any distant metastasis; (2) synchronous tumors or previous treatment; (3) any medical condition incompatible with surgery under general anesthesia or with ICT.

Two homogeneous groups were selected, and both groups were statistically comparable, according to gender, age, tumor location, T stage, N stage, clinical stage, alcohol use, and tobacco use. The T stage and N stage was assessed by physical examination, computed tomography (CT) (or magnetic resonance imaging, MRI). Organ‐preservation group: patients treated with an organ‐preservation protocol consisting of ICT followed by radiotherapy (RT), definitive concurrent chemo‐radiotherapy (CRT), or surgery according to the response rate. Surgery group: patients treated with total laryngectomy, partial pharyngectomy, with (or without) neck dissection initially. The patients chose the treatment voluntarily according to their willingness.

In the organ‐preservation group, the ICT consisted of two 21‐day cycles of docetaxel (75 mg/m^2^ i.v. infusion on Day 1), cisplatin (30 mg/m^2^ i.v. infusion per day from Day 2 to Day 4), and 5‐fluorouracil (500 mg/m^2^ i.v. infusion per day from Day 2 to Day 6). All patients were examined by CT or MRI 3 weeks after ICT to measure the tumor response. Tumor response was defined according to the Response Evaluation Criteria In Solid Tumors (RECIST) criteria.^8^ Responders (complete response, CR or partial response, PR) underwent definitive RT, CRT, or surgery followed by RT/CRT. The regional lymph nodes were assessed separately for the primary tumor. Three months after the end of RT/CRT, patients with residual disease in the lymph nodes were referred for neck dissection. Nonresponders (stable disease, SD or progressive disease, PD) underwent salvage surgery followed by RT/CRT. A multidisciplinary team (MDT) approach was provided during the entire therapeutic period to treat the adverse effects and guarantee the best oncological outcome.

### 2.2 Assessment

In the organ‐preservation group, acute and late treatment‐related toxicities were recorded according to the National Cancer Institute Common Toxicity Criteria for Adverse Events version 3.0. and the Radiation Therapy Oncology Group (RTOG).^9^ PFS is defined as the time in months from the date of treatment to disease progression or death from any cause. OS is defined as the time in months from the date of treatment to death (or date of last follow‐up). LFPS is defined as survival without a tracheotomy, feeding tube, or local disease evolution.

### 2.3 Statistical analysis

SPSS Statistics software (IBM v 23.0) was used for the statistical analysis. The chi‐square test was used for the analysis of categorical variables. The Kaplan–Meier method was used for the calculation of survival and tested by a log‐rank test. Cox's regression model was used for the multivariate analysis. Confidence intervals (CIs) were presented at the two‐sided 95% confidence level and the level of significance was set at *p* < 0.05.

## 3 RESULTS

### 3.1 Patient characteristics

**FIGURE 1:**
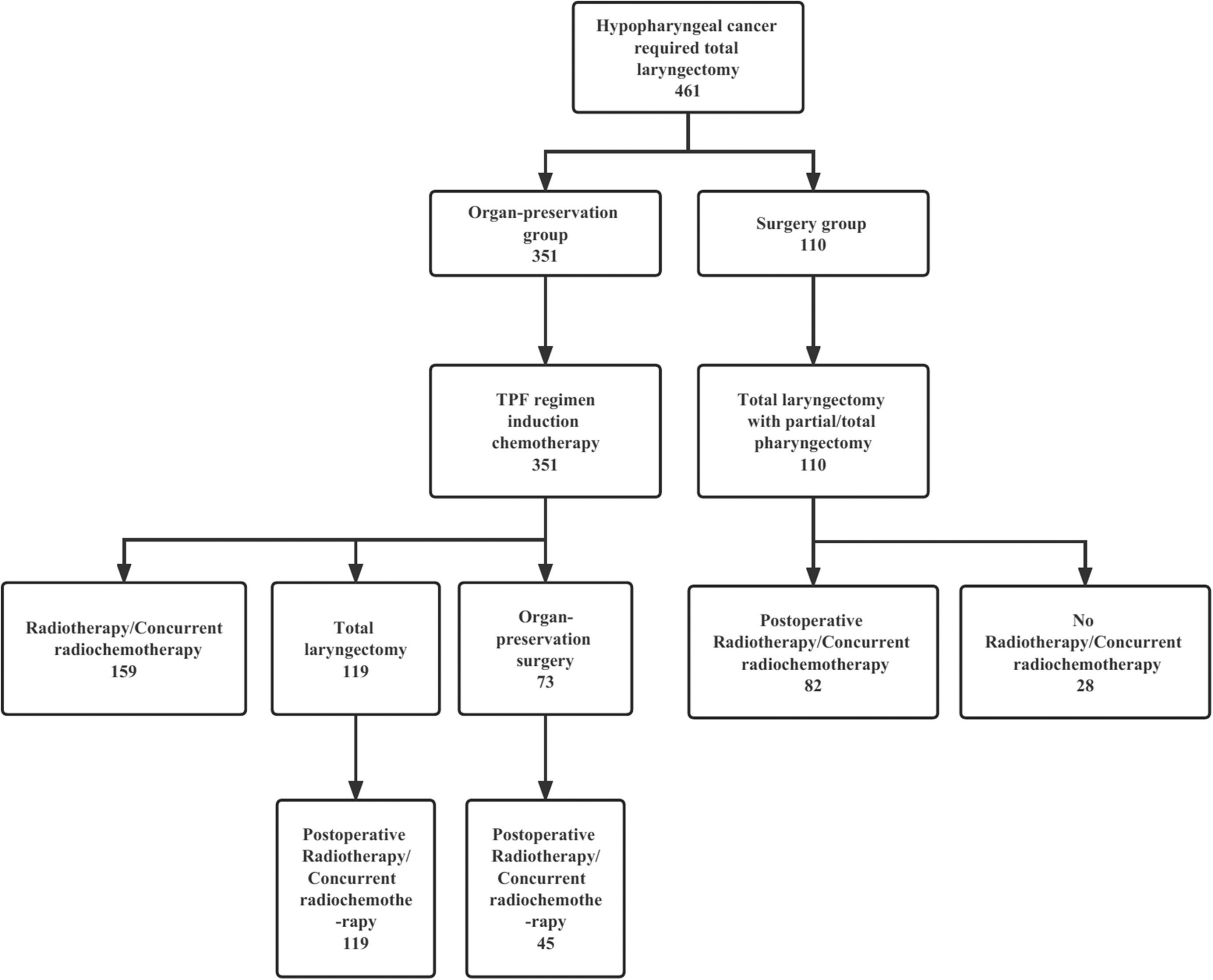
A concert diagram for each arm describing the management of the patients.

Table 1 shows the patients' characteristics. In the organ‐preservation group, the mean age was 57.42 ± 7.75 years (range, 35 to 78 years) and in the surgery group, the mean age was 60.40 ± 9.70 years (range, 35 to 79 years). The baseline characteristics were well balanced between the two groups according to age, gender, tobacco use, alcohol use, tumor location, T stage, N stage, and clinical stage (*p* > 0.05). The follow‐up time ranged from 6 to 228 months. Figure 1 showed the concert diagram for each arm.

**TABLE 1:** Baseline characteristics of the two groups.

Characteristics	Group		*p*
	Organ‐preservation group (*n* = 351)	Surgery group (*n* = 110)	
Age			
<55 years	129	37	0.552
≥55 years	222	73	
Gender			
Male	341	106	0.675
Female	10	4	
Tobacco			
No‐light smoker	289	84	0.164
Moderate‐heavy smoker	62	26	
Alcohol			
No‐light drinker	251	69	0.081
Moderate‐heavy drinker	100	41	
Location			
Pyriform sinus	272	80	0.064
Retropharyngeal wall	51	13	
Postcricoid region	28	17	
T stage			
T2	30	5	0.075
T3	136	55	
T4	185	50	
N stage			
N0	69	26	0.248
N1	59	23	
N2	219	58	
N3	4	3	
Clinical stage			
III	62	27	0.111
IV	289	83	

### 3.2 Organ‐preservation group

All 351 patients in this group received two cycles of ICT. Out of 351 patients, 41 patients (11.7%) had a CR at the primary tumor site. Then the patients proceed to definitive RT or CRT. 225 patients (64.1%) had a PR at the primary tumor site. Then the patients proceed to definitive RT, CRT, organ‐preservation surgery, or total laryngectomy followed by RT/CRT according to the MDT. Sixty‐two patients (17.7%) experienced a SD. Twenty‐three patients (6.6%) experienced a PD and proceed to salvage surgery followed by RT/CRT.

Organ‐preservation surgery was proposed for 73 patients (20.8%). Of the 73 patients, 26 patients (35.6%) received postoperative RT, 19 patients (26.0%) received postoperative CRT. Total laryngectomy was performed for 119 patients (33.9%). Of the 119 patients, 97 patients (81.5%) received postoperative RT, 22 patients (18.5%) received postoperative CRT. During the follow‐up, 116 patients (33.0%) presented locoregional recurrence. Forty‐nine patients (14.0%) presented distant metastases. The most frequent location was the lung (29 patients), followed by the bone (7 patients), liver (7 patients), and brain (6 patients). Thirty‐nine patients (11.1%) presented a second primary tumor. The most frequent location was the esophagus (26 patients), followed by the lung (5 patients), tongue (4 patients), colorectal (2 patients), gastric (1 patient), and ovarian (1 patient).

In the surgery group, 26 of 116 patients with a locoregional failure were treated by chemotherapy and 22 by reirradiation/chemoradiation (0 were controlled), 5 only had tracheotomy, 3 had microlaryngeal surgery, 33 had salvage surgery (effective in 10), 17 had supportive care only, and 10 had no treatment. Among HPC patients who developed distant metastases, no one was controlled.

During the TPF ICT, 273 patients (77.8%) experienced some type of toxicity. The most frequent toxicities were hematologic toxicity (238 patients, 67.8% of all), nausea and/or vomiting (193 patients, 55.0% of all), diarrhea (37 patients, 10.5%), abnormal liver function (31 patients, 8.8%), arthralgia (8 patients, 2.3%), and febrile neutropenia (5 patients, 1.4%). No Grade 4 toxicity was occurred.

### 3.3 Surgery group

All 110 patients in this group underwent the total laryngectomy with partial/total pharyngectomy. Among them, 94 patients (85.5%) underwent the neck dissection simultaneously. Eighty‐two patients (74.5%) received postoperative RT or CRT. During the follow‐up, 39 patients (35.5%) presented locoregional recurrence. Twenty patients (18.2%) presented distant metastases. The most frequent location was the lung (11 patients), followed by the bone (5 patients), brain (2 patients), and liver (2 patients). Nine patients (8.2%) presented a second primary tumor. The most common location was the esophagus (4 patients), the next was the lung (3 patients), and tongue (2 patients).

In the surgery group, 14 of 39 patients with a locoregional failure were treated by chemotherapy and 6 by reirradiation (1 was under control), 2 had tracheotomy, 1 had microlaryngeal surgery, 6 had salvage surgery (effective in 4), 7 had no treatment, 3 had supportive care only. Among HPC patients who developed distant metastases, no one was controlled.

### 3.4 Survival analysis

The 3‐, 5‐, and 10‐year OS rates were 42.5% (95% CI 36.8% to 48.2%), 32.5% (95% CI 27.0% to 38.0%), and 23.2% (95% CI 17.1% to 29.3%) in the organ‐preservation group and 43.8% (95% CI 33.4% to 54.2%), 29.2% (95% CI 19.0% to 39.4%), and 16.4% (95% CI 7.2% to 25.6%) in the surgery group, respectively.

The 3‐, 5‐, and 10‐year PFS rates were 36.5% (95% CI 31.0% to 42.0%), 29.5% (95% CI 24.2% to 34.8%), and 22.0% (95% CI 16.1% to 27.9%) in the organ‐preservation group and 39.2% (95% CI 29.2% to 49.2%), 28.3% (95% CI 18.3% to 38.3%), and 12.2% (95% CI 4.0% to 20.4%) in the surgery group, respectively.

**FIGURE 2:**
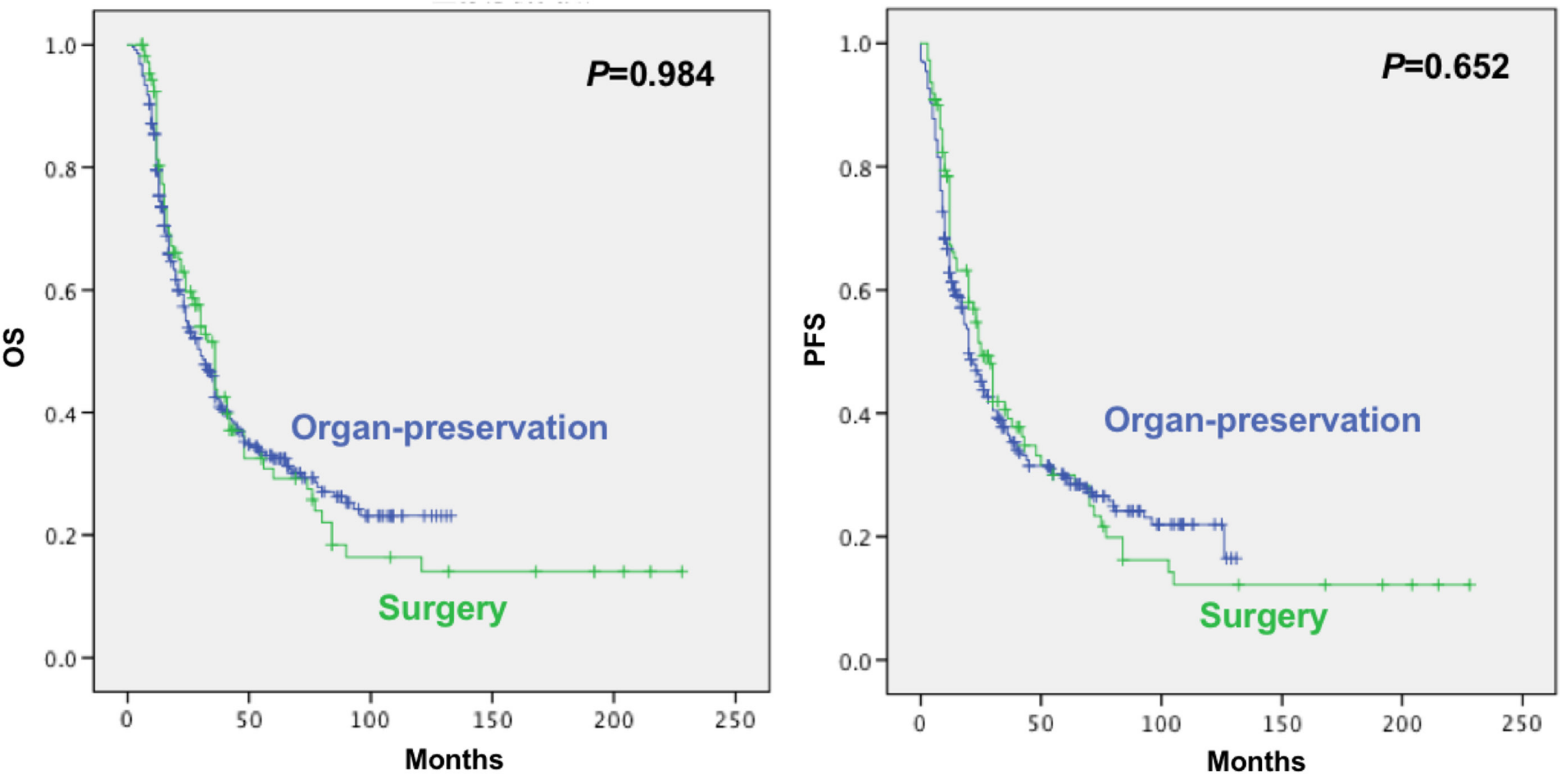
Comparison of overall survival and progression‐free survival data between the surgery and organ‐preservation groups.

Log‐rank analysis did not show a significant difference in OS and PFS between both groups (all *p* values >0.05) (Figure 2).

**FIGURE 3:**
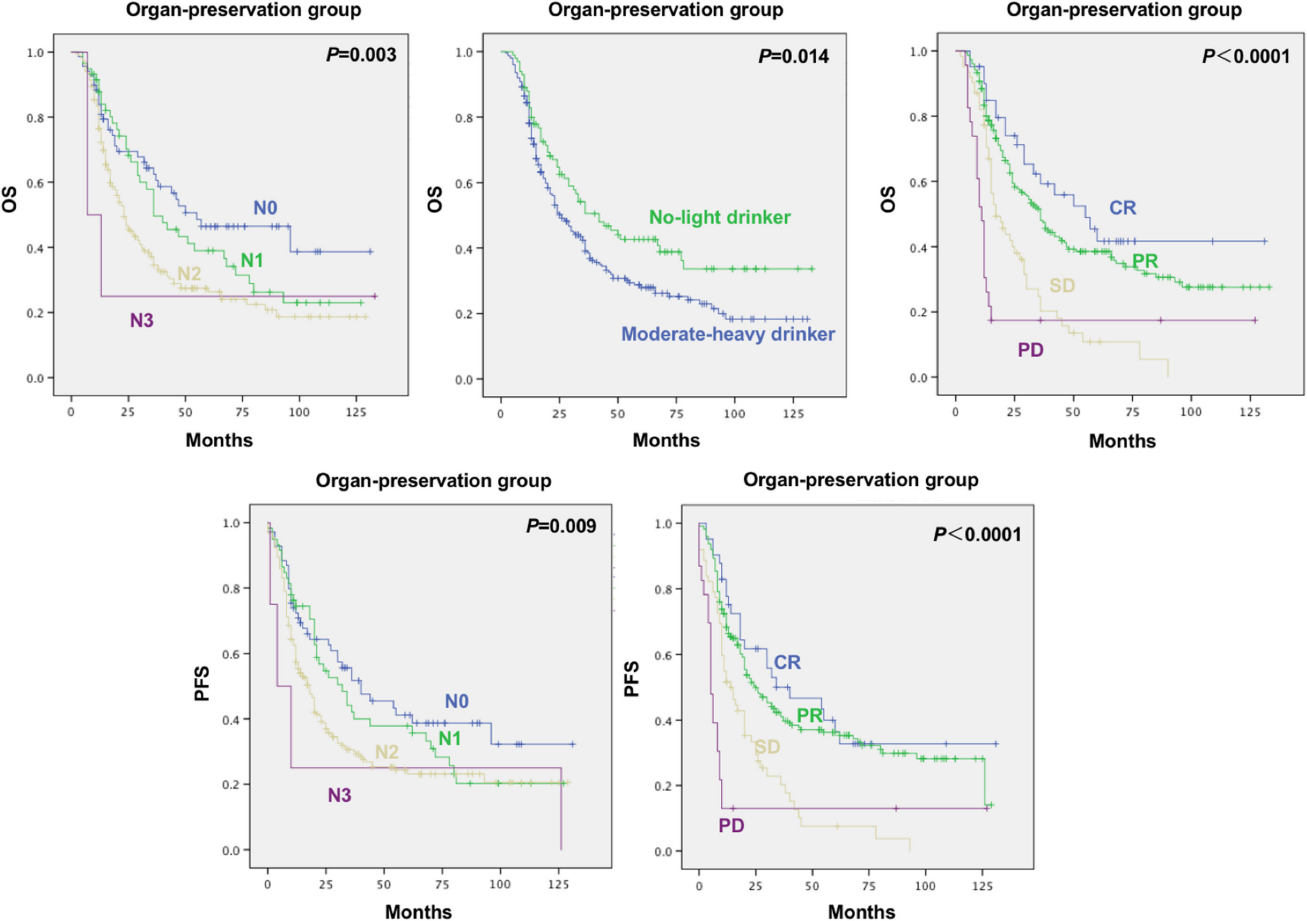
Comparison of overall survival and progression‐free survival data in the organ‐preservation group, stratified by N stage, alcohol consumer, and response rate.

We observed that the N stage, and the tumor response after ICT had significant prognostic impacts for the OS and PFS. The OS of N0 > N1 > N2 > N3 patients (*p* = 0.003), the OS of CR > PR > SD > PD patients (*p* < 0.0001). The PFS of N0 > N1 > N2 > N3 patients (*p* = 0.009), the PFS of CR > PR > SD > PD patients (*p* < 0.0001). Moreover, the OS of no‐light drinkers>moderate‐heavy drinkers (*p* = 0.014) (Figure 3). (Moderate drinker—More than 3 drinks but no more than 7 drinks per week for women and more than 3 drinks but no more than 14 drinks per week for men, on average over the past year. Heavy drinker—More than 7 drinks per week for women; more than 14 drinks per week for men, on average over the past year. Moderate smoker—16 to 24 cigarettes per day. Heavy smoker—≥25 cigarettes per day.)

While we found that in the surgery group, the N stage had significant prognostic impact for the OS and PFS. The OS of N0 > N1 > N2 > N3 patients (*p* = 0.031). The PFS of N0 > N1 > N2 > N3 patients (*p* = 0.04) (Figure S1).

When the analyses stratified by the T stage, N stage, and clinical stage, there were no significant differences in OS and PFS between the organ‐preservation group and the surgery group (all *p* values >0.05) (Figure S2).

In the organ‐preservation group, the 3‐, and 5‐, and 10‐year LFPS rate were 30.7% (95% CI 25.6% to 35.8%), 23.2% (95% CI 18.3% to 28.1%), and 16.6% (95% CI 11.3% to 21.9%). We observed that the T stage, N stage, clinical stage, and the tumor response after ICT had significant prognostic impacts for the LFPS. The LFPS of T2 > T3 > T4 patients (*p* = 0.002), N0 > N1 > N2 > N3 patients (*p* = 0.019), Stage III > Stage IV patients (*p* = 0.001), CR > PR > SD > PD patients (*p* < 0.0001) (Figure S3).

Table 2 shows that compared with surgery group, organ‐preservation group was uncorrelated with an ascending risk of disease progression and patient death.

**TABLE 2:** Hazard ratios for event rate associated with ICT based organ‐preservation therapy.

Cox's regression analysis on matching variables	Value
Overall survival	
Hazard ratio	1.003
95% CI	0.77–1.31
*p*	0.98
Progression‐free survival	
Hazard ratio	0.943
95% CI	0.73–1.22
*p*	0.66

## 4 DISCUSSION

HPSCC has one of the worst prognoses among head and neck malignancies, with a 5‐year OS rate of less than 35%.^10^ Until the early 1990s, the main therapeutic modality for HPSCC who are unsuitable for laryngeal function preservation surgery was total laryngectomy. The fearful surgery brought crushing effects to patients including loss of speech sounds, loss their job, unaesthetic appearance, depression, and social isolation. Recently, the overarching goal is to optimize survival and improve organ function preservation, and various nonsurgical approaches for organ preservation have been widely used. Patients with locally advanced HPC who need total laryngectomy but for whom larynx function is indispensable can be considered with these approaches. It is vital to understand when best to apply organ preservation strategies. According to European Society of Medical Oncology guidelines in 2020,^11^ in HPSCC patients requiring total laryngectomy without laryngeal cartilage invasion, TPF ICT followed by RT in case of complete or partial response and concomitant CRT are options for function preservation. According to National Comprehensive Cancer Network (NCCN) guidelines published in 2022,^12^ the organ preservation options for T2‐3, N0‐3 HPSCC patients requiring total laryngectomy are ICT followed by RT/systemic therapy/surgery (in case of complete or partial response) or concurrent systemic therapy/RT. T4a, N0‐3 patients who decline surgery should consider ICT followed by RT/systemic therapy (in case of complete or partial response) or concurrent systemic therapy/RT.

CCRT and ICT followed by comprehensive therapy are two recommended approaches available for larynx preservation. Platinum‐based CCRT is a valid option for organ preservation and has emerged as the treatment choice.^13^ Despite the incontestable advantages, CCRT is associated with acute and late toxicities, which may lead to increased noncancer‐related morbidity, organ failures, unplanned interruptions, and influencing salvage surgery.^14^ Due to the treatment‐related toxicity, CCRT should be reserved for selected patients and requires expertise in oncological focus more on supportive care. According to the meta‐analysis, patients who received TPF experienced lower frequencies of nausea, stomatitis, vomiting, hearing loss, and grade 3/4 mucositis.^15^ Compared with CCRT, ICT might enhance efficacy by selecting patients earlier. The ICT does not compromise subsequent CRT or surgery. In nonresponders, salvage surgery could be performed immediately before CCRT/RT. Patient choices are also important in treatment decisions. In China, some HPSCC patients prefer to leave some salvage opportunities for their future treatment. They are more likely to accept the strategy of ICT to preserve their organ function and decide the next strategy by observing the treatment response. Some patients who are afraid to experience the underlying toxicities of ICT/CRT without any earning would choose to receive an initial total laryngectomy. Knowledge of the systemic consequences related to treatment strategy together with the conditions of the patients at baseline are of crucial importance. Therefore, we advocated that MDT management approach should be provided during the entire therapeutic period. Combining these factors, we conducted an analysis to compare the ICT‐based organ‐preservation protocol and immediate total laryngectomy for locally advanced HPSCC. In the ICT‐based group, the therapy was quite well tolerated, no severe toxic effect (Grade 4 toxicity) was occurred.

Our result revealed no significant differences in 3‐, 5‐, and 10‐year OS and PFS between the organ‐preservation group and the surgery group. Although PFS is not a perfect comparison in organ preservation versus laryngectomy study, it is a relatively good indicator to compare the treatment effect. In NCCN guidelines, for T2‐3, N0‐3 HPSCC patients, the first recommended therapy is ICT, and the second recommended therapy is partial or total laryngopharyngectomy. On the contrary, for T4a, N0‐3 patients, the first recommended therapy is total laryngopharyngectomy, and the second recommended therapy is ICT. Interestingly, in our stratification analysis by T stage, similar OS and PFS were observed for T2‐stage patients between the organ‐preservation group and the surgery group. The results of stratification analysis for T3‐stage or T4‐stage patients were the same. This may indicate that no matter whether for T2, T3, or T4 resectable HPSCC patients, the survival data were not related to the choice of ICT or initial surgery.

Most HPSCC patients develop lymph node metastasis at initial diagnosis.^16^ In our study, the poor OS and PFS of the two groups were all related to the advanced N stage. Comprehension the schema of lymph node metastasis may help both patients and surgeons predict the prognosis. Whether a more radical lymph node dissection regimen can help improve the survival of patients is a question worth studying in the future. We noticed that in the organ‐preservation group, according to the alcohol consumption, moderate‐heavy drinkers had a worse prognosis than no‐light drinkers. Alcohol has been classified as a carcinogen (Class I) by the World Health Organization. Evidence is accumulating that alcohol consumption is causally related to head and neck cancer.^17^ A recent study demonstrated that temperance in heavy‐moderate drinkers refines cancer‐related growth factors. The sudden reduce in serum EGF and VEGF was found in 90% of patients in the temperance group.^18^ For the common population, drinking less alcohol is a healthy lifestyle. For HPSCC patients, moderate‐heavy alcohol consumption history may negatively affect the OS of an ICT‐based organ‐preservation strategy, which is a reminder of treatment choices. Our future research will further investigate the molecular mechanism of this phenomenon.

The response of ICT may predict the sensitivity of RT and CCRT.^19^ In our study, we found that better tumor response after ICT had significantly better prognostic impacts for the OS and PFS. CR patients had the best survival benefits, followed by PR patients, which means the response of ICT may select patients earlier and predict the prognosis of patients. In nonresponders, surgery could be conducted in time before CCRT/RT. The better tumor response could also indicate a better LFPS. Moreover, in the organ‐preservation group, the LFPS were negatively correlated with the grade of clinical stage, N stage, and T stage. The earlier the stage, the better organ function will be preserved.

We observed that in our surgery group, the 10‐year OS and PFS rate were 16.4% and 12.2%, respectively, which were comparable with the results of EORTC trial 24,891 (10‐year OS and PFS rate were 13.8% and 8.5%, respectively). However, in our organ‐preservation group, the 10‐year OS and PFS rate were 23.2% and 22.0%, respectively, which seemed higher than the results of EORTC trial 24,891 (10‐year OS and PFS rate were 13.1% and 10.8%, respectively).^5^ Because of the nature of retrospective and nonrandomized research, our results were biased by the selection function of ICT. Patients who were nonresponders of ICT tended to give up the subsequent therapy and had only supportive care (excluding from our study). This favored the survival outcome of the organ‐preservation group because nonresponders were likely to have a poorer prognosis. This might be one of the limitations of the study. In the future, prospective, multicenter, and larger studies will be adopted to maximize research accuracy. Another main limitation was the variation protocol of RT. Our research was from 2002 to 2022, the intensity‐modulated radiotherapy (IMRT) has been considered the gold standard instead of two‐dimensional RT. Moreover, recent studies on volumetric modulated arc therapy (VMAT) showed some advantages at target coverage in organ and risk sharing when in comparison with IMRT.^20^ Therefore, the RT scheme was not uniform in our study. Matched‐pair analysis is unable to substitute prospective and randomized studies, and standard RT regimens need to eliminate the biases, but in the absence of such a study, it is one of the best options. In some study, the TPF regimen was given more than two cycles, imaging is required after two cycles of chemotherapy to reevaluate tumor changes and to develop further treatment plans based on the changes. This may not only increase the efficacy of ICT, but also increase the toxic side effects. However, there is no exact requirement of how many cycles are needed. In the future, the combination of ICT, targeting therapy, and immunotherapy are expected to improve the prognosis of locally advanced HPSCC. Further investigation of intra‐tumoral heterogeneity, tumor microenvironment, and molecular mechanism in tumor regulation would help to find high‐efficient treatment strategies and select proper patients to apply organ‐preservation approaches.

In conclusion, our results showed the safety and feasibility of ICT‐based organ‐preservation protocol in HPSCC requiring total laryngectomy. There was no significant difference between the two groups related to survival outcomes. The organ‐preservation approach could improve the LFPS without compromising the OS and PFS, and allowed more than 70% of the survivors to retain their larynx function. However, the treatment choices involve comprehensive factors, the MDT approach is recommended to guarantee the best oncological outcome individually.

## AUTHOR CONTRIBUTIONS

**Yifan Yang:** Conceptualization (lead); data curation (lead); formal analysis (lead); investigation (lead); methodology (lead); software (lead); visualization (lead); writing – original draft (lead). **Ling Feng:** Conceptualization (equal); data curation (equal); methodology (equal); resources (equal); supervision (equal); writing – review and editing (equal). **QI ZHONG:** Conceptualization (equal); project administration (equal); resources (equal); supervision (equal); validation (equal). **Yang Zhang:** Resources (equal); supervision (equal); validation (equal). **zhigang huang:** Resources (equal); supervision (equal); validation (equal). **Shurong Zhang:** Investigation (equal); methodology (equal); resources (equal); supervision (equal); validation (equal). **Shuling Li:** Methodology (equal); resources (equal); validation (equal). **Junmao Gao:** Investigation (equal); methodology (equal); resources (equal); supervision (equal); validation (equal). **Lizhen Hou:** Investigation (equal); methodology (equal); resources (equal); supervision (equal). **Hongzhi Ma:** Investigation (equal); methodology (equal); resources (equal); validation (equal). **Shizhi He:** Investigation (equal); methodology (equal); software (equal); validation (equal). **Qian Shi:** Funding acquisition (equal); investigation (equal); resources (equal); visualization (equal). **Meng Lian:** Data curation (equal); investigation (equal); resources (equal); visualization (equal). **Yanming Zhao:** Formal analysis (equal); investigation (equal); resources (equal); software (equal). **Xixi Shen:** Investigation (equal); methodology (equal). **Jiaming Chen:** Investigation (equal); software (equal). **Lingwa Wang:** Investigation (equal); software (equal). **Haiyang Li:** Software (equal); visualization (equal). **Shaoshi Chen:** Investigation (equal); software (equal). **Jiaqi Xu:** Investigation (equal); software (equal). **Ru Wang:** Data curation (equal); funding acquisition (equal); project administration (equal); supervision (equal); validation (equal). **Jugao Fang:** Conceptualization (equal); funding acquisition (lead); methodology (equal); project administration (lead); resources (lead); supervision (lead); validation (lead); writing – review and editing (lead).

## CONFLICT OF INTEREST STATEMENT

The authors have no conflict of interest to declare.

## Supporting information


Figure S1.

Figure S2.

Figure S3.

## Data Availability

The datasets generated during and/or analyzed during the current study are available in the manuscript.
