# Supplementary figures and images for: Induction chemotherapy‐based organ‐preservation protocol improve the function preservation compared with immediate total laryngectomy for locally advanced hypopharyngeal cancer—Results of a matched‐pair analysis
Source: Cancer Med. 2023 Jul 19;12(16):17078–86. doi: 10.1002/cam4.6354 (PMC10501291; doi:10.1002/cam4.6354)

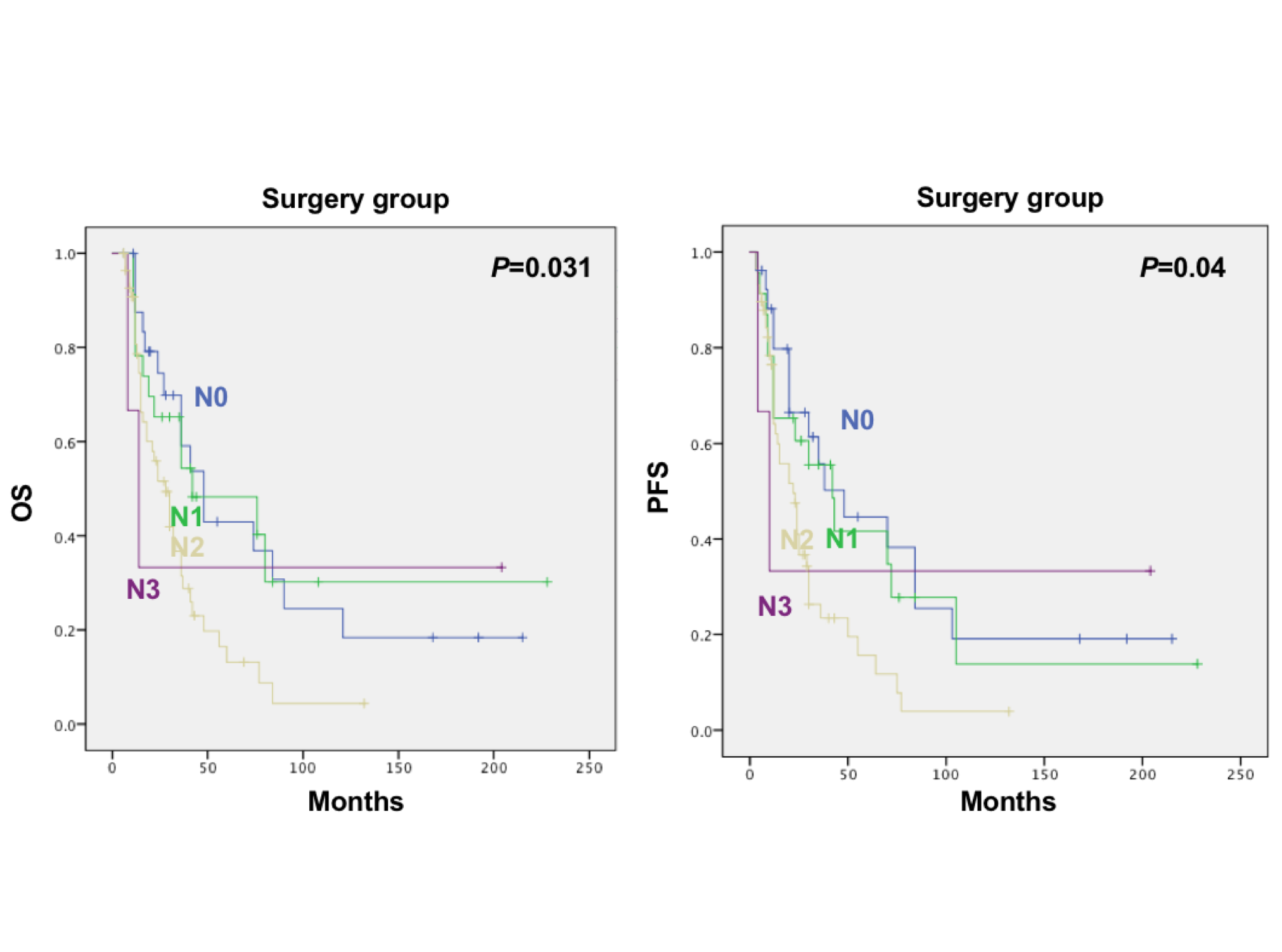

Supplement: Supplementary file 1 — Figure S1. [file CAM4-12-17078-s002.tiff]

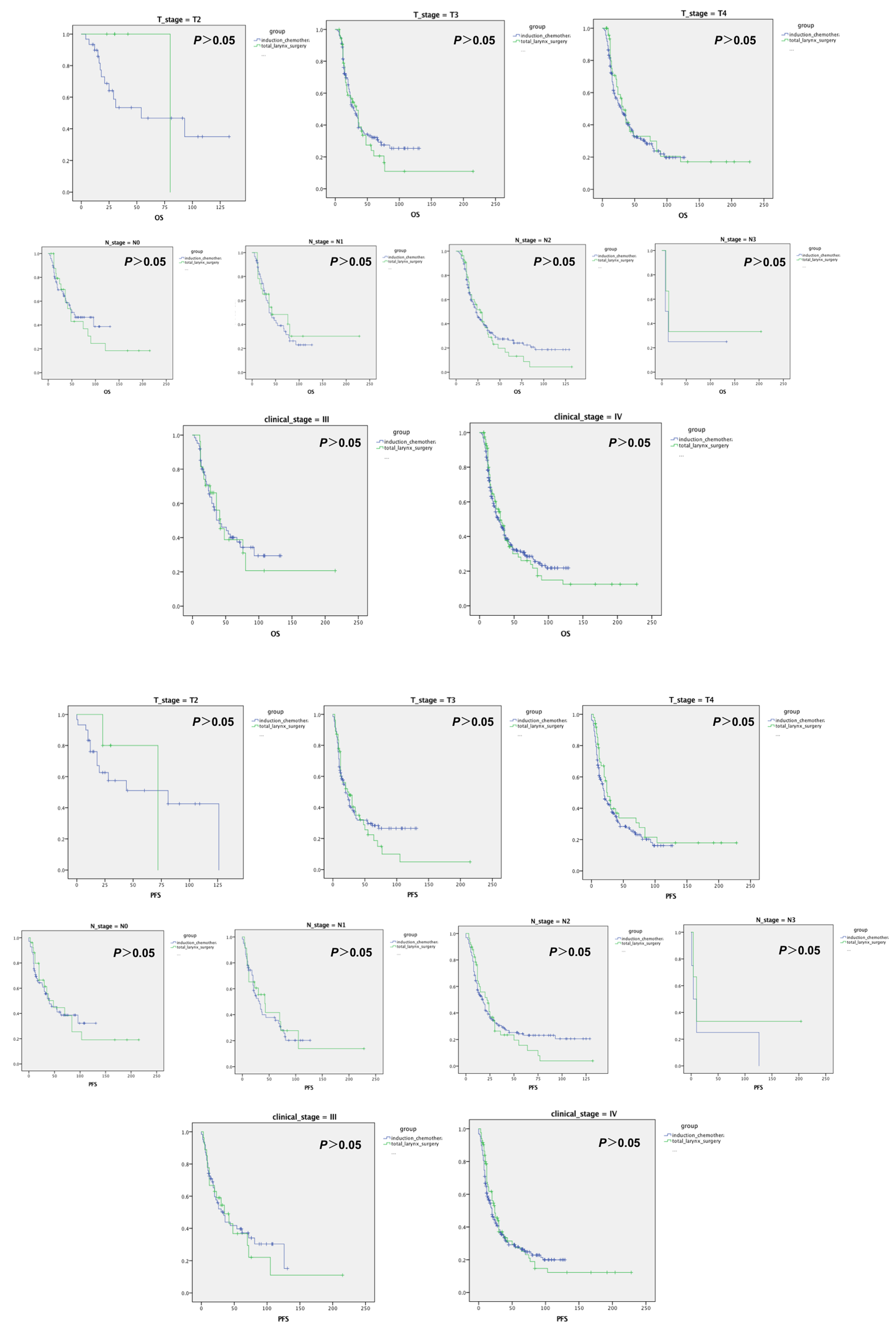

Supplement: Supplementary file 2 — Figure S2. [file CAM4-12-17078-s003.tif]

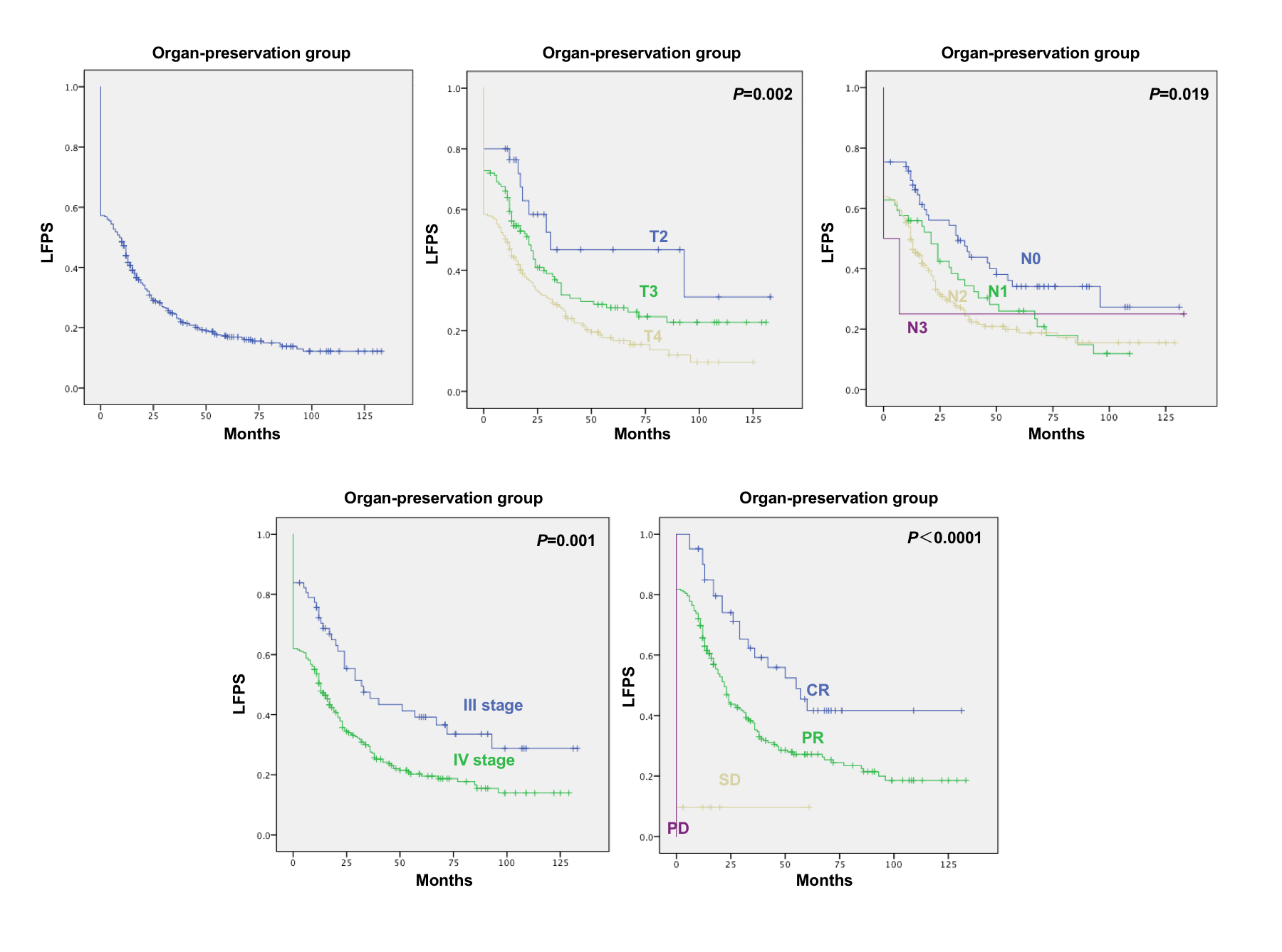

Supplement: Supplementary file 3 — Figure S3. [file CAM4-12-17078-s001.tiff]
